# En Route to Midwifery Professionalisation: Contextualising Midwifery Care in the Indian Public Health System

**DOI:** 10.1177/2752535X251340760

**Published:** 2025-05-11

**Authors:** Sanjana Santosh

**Affiliations:** 1 Nossal Institute for Global Health, Melbourne School of Population and Global Health, 549317 University of Melbourne, Parkville, VIC, Australia

**Keywords:** Health Policy, Professional Boundaries, Maternal Health, Professionalisation, Midwifery

## Abstract

**Background:**

The ‘Guidelines on Midwifery Services Initiative, 2018’ introduces the Nurse Practitioner in Midwifery (NPM) cadre to improve maternal mortality rates, quality of care, and reduce overmedicalisation in the Indian public health system. The paper examines how the NPM cadre is envisioned in the guidelines, positioned within the medical hierarchy, and aligned within the existing maternal health context. It also looks at how existing midwives and midwifery advocates perceive the changes introduced and the accompanying professionalisation process.

**Methodology:**

The study employed two methods: (a) qualitative document analysis (QDA) of the midwifery guidelines and (b) semi-structured in-depth interviews with 14 nurse-midwifery leaders and advocates.

**Findings:**

The QDA uncovers the underlying themes of task shifting and institutional deliveries as dominant, discussions on professional autonomy and primary health care receiving less importance and even less reflection on the history of midwifery in India. The interview findings highlight (a) tensions around carving out a separate profession, (b) the implications of midwifery at the primary healthcare level and (c) the need for an enabling environment for midwives. This paper reveals how the guidelines frame midwifery as a cost-effective, specialised nursing care within the institutional delivery framework and distanced from its traditional connotations.

**Discussion:**

It elucidates tensions around autonomous midwifery involving professional boundaries, negotiations with medical professionals, institutional perceptions, and historical stereotypes. It builds on the broader literature on ‘professions’ in sociology by identifying aspects of ‘professional boundary work’ embedded in policy documents and experiences of participants – thereby unpacking the midwifery professionalisation process in the Indian context.

## Introduction

National Family Health Survey-5 data shows a rise in caesarean section rates from 8.5% in 2005-06 to 21.5% in 2019-21, with higher rates in the private sector and significant state-wise variation, from 5.2% in Nagaland to 60.7% in Telangana.^1–3^ Addressing concerns regarding the over-medicalisation of childbirth, the Government of India introduced the Nurse Practitioner in Midwifery (NPM) cadre through the 2018 ‘Guidelines on Midwifery Services Initiative.’ Midwifery is increasingly recognised as a solution to reduce maternal mortality and medical interventions while enhancing access to sexual, reproductive, maternal and newborn child health (SRMNC).^4,5^ This initiative builds midwifery competencies through an 18-month training program for nurses with a General Nurse Midwifery (GNM) degree or BSc. Nursing degree and 2 years of clinical experience, converting them into professional Nurse Practitioners in Midwifery (NPM).^6^

Since introducing these guidelines, states like Telangana, Maharashtra, Bihar, and Karnataka have launched midwifery training programs. Private hospitals following international midwifery standards and collaborating with international midwives have partnered and supported implementing some of these programs.^7^ During the study period (2020-2021), NPM and midwifery trainer training facilities were being established, and the first batch of NPMs was integrated into the public health system in Telangana, a state with one of the highest C-section rates in India.^3^ This study, conducted during the initial uptake of midwifery programs, explores policy conceptualisation and the professionalisation process of the midwifery cadre without evaluating midwifery practice.

While recruiting midwives is crucial for addressing SRMNC health inequities, “midwifery cannot offer an effective alternative without sufficient understanding of its roots of injustice.”^8(p.356)^ The history of midwifery in India involves casteist practices of ritual pollution, colonial efforts to modernise childbirth care, the dominance of hospital-based obstetric models, and the subordination of midwifery within the nurse-midwifery practice.^9–11^ This study examines recent policy to professionalise midwifery within India’s historical and current context of maternal healthcare.

The professionalisation of midwifery, and professionalisation in general, involves constructing and claiming professional jurisdiction while engaging with dominant paradigms and defending against competing claims from neighbouring professions.^8,12,13^ The literature on ‘professions’ in sociology provides insights that help unpack the process of professionalising midwifery in India. The first section outlines the context of midwifery in India and the emerging research questions, followed by methodology, findings, and discussion. While the context of midwifery in India resists generalisation, I offer a brief overview of the key forces that have shaped its trajectory, acknowledging the significant nuance, detail, and history that lie beyond this summary.

## The Context of Midwifery in India

Increasingly, midwifery is recognised for reducing medical interventions and addressing the over-medicalisation of childbirth, provided it is delivered by qualified midwives.^14,15^ Multiple factors, including past policies and how they valued midwifery as a profession, determine the success of integrating midwifery into the health system.^16^ Therefore, understanding its integration within the Indian health system requires examining its broader context and policy legacies.

Feminist scholarship critiques the medicalisation of childbirth, highlighting the dominance of medical technology and obstetric care.^17–19^ In the context of colonisation, scholars argue that this dominance of Western medical knowledge, reinforced by the endorsement by Indian elites, contributed to the rationalisation and vilification of local birth practices and Indian traditional midwives, or ‘Dais’.^10,20,21^ Scholars have documented colonial descriptions of ‘dais’ as ‘unhygienic and barbaric’ and their marginalisation due to casteist cultural notions of ritual pollution.^9,22,23^

Although the role of traditional midwifery was acknowledged in reducing maternal mortality in rural areas, colonial and postcolonial training attempts resulted in bundling varied local healers as Traditional Birth Attendants (TBAs) without effective knowledge exchange, prescribing biomedical training that ultimately rendered them ineffective.^24–26^ Following this, the investment in TBA training was limited due to its high resource demands and minimal impact on maternal mortality, leading to a preference for training existing nursing cadres as professional midwives.^11,27,28^

Historically, nursing and midwifery within the formal health system have been limited to subsidiary roles, and furthermore, midwifery has been a secondary component within the nursing profession. While British nurses and midwives were distinct professionals, and colonial efforts in building nursing and midwifery cadres in India also had separate midwifery trainings, post-independence, the regulatory reforms establishing the Indian Nursing Council initiated the unification of nursing and midwifery. The Council recognised only two integrated training programs – General Nursing and Midwifery (GNM) and Auxiliary Nurse Midwifery (ANM), creating a joint registered nurse and registered midwife cadre (RNRM).^11,29^ Scholars trace the historical trajectory of nursing, revealing that the positions of Registered Nurses and Registered Midwives (RNRMs) were seldom held by upper-caste or upper-class women due to casteist stigma. Post-independence, the Indian government faced challenges in attracting educated women from ‘good families’ to the nursing profession.^30–32^ Until recently, India lacked a nationwide independent midwifery cadre, as a ‘midwife’ could be an Auxiliary Nurse and Midwife (ANM) or a Staff Nurse (SN). It was further observed that the practical training and scope for midwifery practice for both ANMs and SNs shrunk over the years, especially for ANMs who were converted into Multi-Purpose Health Workers (MPHWs) catering to vertical disease programmes.^33,34^ We do see attempts to introduce the NPM cadre in the early 2000s, but they were short-lived, fragmented and eventually discontinued.^6(p.4)^

The dilution of the midwifery component within nursing must also be understood in the larger neoliberal direction of the global health policy discussions and the influence it extended in local contexts.^35^ Despite the Alma-Ata declaration’s focus on primary health care, financial limitations in the Indian context shaped its adoption of vertical health programs over comprehensive primary care.^36,37^ The Millennium Development Goals’ emphasis on reducing maternal mortality, coupled with the promotion of institutional births, further marginalised the role of midwives.^11,38^ The push for institutional births at both international and national levels, aiming to increase ‘safe deliveries,’ inadvertently reinforced technocratic values and prioritised efficiency.

Institutional delivery policies have shown positive results in reducing maternal mortality, with India’s Maternal Mortality Ratio (MMR) at 97. However, there are variations in region and caste, as certain states and caste groups have higher maternal mortality rates.^39,40^ Effective maternal health programs require improvements in quality and equity, addressing issues like informal payments (money paid to hospital staff for better care and help) and out-of-pocket expenses at tertiary institutions. Well-equipped primary health centres (PHCs) are critical for delivering high-quality maternal health services. Despite increased maternal morbidities associated with poorly equipped PHCs, women demonstrated a clear preference for well-equipped PHCs when available.^41–46^ Moreover, increased workload and congestion in tertiary health facilities have been shown to contribute to over-medicalisation, obstetric violence, and disrespect during childbirth.^47,48^

The recent emphasis on respectful maternity care and midwifery’s role in reducing medicalisation has brought midwifery back into policy discussions.^49^ The 2018 Guidelines on Midwifery Services Initiative in India highlight the need for de-medicalization, stating that ‘midwifery care introduces a system-level shift from fragmented maternal and newborn care focused on identification and treatment of pathology to skilled and compassionate woman-centric care.’^6^ A quick review of the guidelines document highlights the involvement of government bodies such as the Ministry of Health and Family Welfare, National Health Mission, State-level Maternal Health Divisions, and the Indian Nursing Council, alongside international organisations like UNICEF, UNFPA, WHO, and private institutions such as Fernandez Hospital and NGOs like Jhpiego. While midwives are present in the contributor’s list, their limited representation underlines the broader challenges in embedding midwifery leadership in policy processes.^50^

Considering the historically and persistently subordinate status of nursing and midwifery in India, it is important to understand the role of the ‘Nurse Practitioner in Midwifery’ cadre as envisioned in the guideline, positioned within the medical hierarchy, and aligned with the existing maternal health context. This study investigates the following research questions-

(1) How does the guidelines propose and frame the need for the ‘nurse practitioner in midwifery’ cadre and structure its role and position within the existing maternal and child healthcare delivery system in India?
(2) How do existing NPMs and midwifery advocates perceive the changes introduced by the guidelines and the accompanying professionalisation process?

## Methodology

### Research Methods

The study mainly employed two methods - qualitative document analysis (QDA) of the guidelines and in-depth interviews. For this study, a document analysis of the ‘Guidelines on Midwifery Services Initiative India, 2018’ was conducted to contextualise the policy document in the larger historical and current Indian maternal healthcare delivery context. Document analysis holds significant value as documents often provide rich insights into the purposes, rationale, and history of programs and events.^51^ It is crucial to recognise that historical facts and evaluations are always mediated through the recorder’s perspective, as ‘the facts of history and evaluation never come to us pure’ and often reflect the context and the position of the actors and institutions involved.^51(p.19-20)^ This necessitates a focus on the selective mechanisms that determine which facts are documented, which are omitted, and how.

Documents are also understood as ‘social facts,’ created, consumed, shared, and utilised in socially organised ways,^52^ and as ‘social artefacts’ produced by actors within specific historical and socio-political contexts.^53^ This study treats the guidelines document as a product of its social environment to uncover the underlying themes, biases, and ideological positions embedded within it.^54^ The QDA conducted here involves examining the text to identify contextual variables, key actors, and the framing of policy issues alongside the dominant, weak, and absent themes.^55,56^ This approach addresses the research question concerning the proposed midwifery model, its rationale, and the envisioned role of the ‘nurse practitioner midwife’ within India’s maternal and child healthcare delivery system.

The second method, the in-depth interviews, as a qualitative research technique, is used to conduct detailed interviews employing an open-ended, discovery-oriented and conversational format, allowing participants to voice their perspectives and experiences.^57^ Here, it aids in building a midwifery standpoint, revealing the changes the guidelines bring within the profession and the experiences of midwives, nurses and other actors involved in the policy-making process. Integrating document analysis with interviews provides valuable insights for policy analysis by offering essential background information and corroborating or challenging interview data.^58^ Document analysis offers a broader contextual understanding by examining texts like policy documents, reports, and media articles, while in-depth interviews provide detailed, personal insights.^55^ QDA situates interview data within a larger historical and social context, deepening the understanding of interviewees’ perspectives.^54^ The two methods complement each other, filling gaps in documents with interview data and vice versa, producing a holistic understanding (See 59–61). Combining these methods enhances the credibility and validity of research findings through triangulation.^62^ Conducted during the early implementation of the NPM program, this approach facilitated ‘responsive interviewing,’ capturing participants’ views and perceptions of the document, the new midwifery cadre, and the meanings they attributed to it.^63^ Drawing on insights from the document analysis, the in-depth interview tool was tailored to each participant’s background. The interviews covered themes such as the aim of the midwifery guideline, participants’ roles and experiences in midwifery advocacy, and the position of NPMs and NPM trainers in the health system etc. (See Supplemental file 1 for topic list)

### Data Collection and Analysis

Between January and May 2021, a total of 14 semi-structured in-depth interviews, including 9 telephonic and 5 Zoom video call interviews, were conducted, lasting an average of one and a half hours, with nurse-midwifery leaders and advocates. All participants were experts in the field and had been directly involved in drafting the midwifery guideline, engaging in policy process meetings, and/or implementing NPM programmes in the country. Participants were identified through the contributors’ list in the guidelines, by attending online midwifery conferences and asking interviewees whom they considered most involved in midwifery policy advocacy. Out of 14, 12 interviews were conducted in English and 2 in Hindi. Table 1 below provides an overview of the participant profiles.

**Table 1.** Participants Profile.

Profile	No. of Participants
**Work experience of participants**	**Total 14**
Nurse-midwifery practitioner leaders with >10 years of experience	4
Nurse-midwifery trainers/educators with >15 years of experience	7
Midwifery advocates with >10 years of experience (other medical professionals, researchers, consultants)	3
**Government and ** **non-** **government** ** positions**	**Total 14**
*Government midwifery advocates*	8
Participants involved directly or influenced the policymaking (participants involved in guideline drafting)	(4)
*Non-* *governmental* * midwifery advocates*	6
Participants involved directly or influenced the policymaking (participants involved in guideline drafting)	(4)
**Context of participants**	**Total 14**
Participants more experienced in the urban context	4
Participants more experienced in the rural context	10

Conducting online interviews during the pandemic presented both opportunities and challenges. Methodologically, it allowed for broader geographic reach and flexibility but also posed risks such as limited rapport-building and potential technical issues. These factors are particularly relevant as the participants were engaged in pandemic work, which could impact the depth of the data collected. To navigate these ethical issues, extra care was taken by offering flexible interview timings, ensuring multiple conversations and clear communication before the interview, obtaining informed consent, and providing ample opportunity for participants to express concerns and ask questions before and during the interviews. The data analysis was performed using Atlas. ti software to analyse both the guidelines and the interview transcripts. The coding process involved assigning summative attributes to data portions that allowed for uncovering and recording interesting and recurring themes.^64^ Descriptive coding is particularly useful in document analysis for summarising critical and emergent topics in the text or data passages, creating a categorised record of the data’s content.^64(p.70-72)^ The coding process began with deductive coding, using three broad themes identified from the context literature review as broad codes – a) the historical position of midwifery, b) the role of midwifery cadre in the health system, and c) larger health system issues in maternal and child health care (MCH). These codes were used with simultaneous open coding to identify new, emergent codes and subcodes relevant to the guideline.

All the interview transcripts were analysed using the principles of thematic analysis.^65^ The themes from the interviews were corroborated with those from the QDA. After rigorously checking the overlaps and distinct themes, a final coding framework was created to code and analyse all the interviews (Refer to Supplemental file 1 for Coding Framework). A flexible coding process allowed for a comprehensive and nuanced analysis of the guidelines and interview data, providing deeper insights into the policy’s context, rationale, and implications. Analytic memo writing was utilised to reflect on the coding process, track the development of patterns and themes, and fine-tune subsequent data gathering.^66(p.72)^

A reflexive approach was pertinent to this study, guided by the positionality of the researcher as an Indian qualitative researcher invested in health policy and systems research. As someone familiar with the Indian healthcare system, I approached this work with a commitment to staying close to the lived realities of midwives and the challenges they face in the system. My engagement with the midwifery profession has been informed by repeated and in-depth conversations with expert midwives, which have provided valuable insights into their experiences and the evolving professionalisation of midwifery in India. In line with Braun and Clarke’s (2022) approach to reflexive thematic analysis, this study adopted an inductive and reflexive analytic stance, grounding the analysis in participants’ narratives while actively constructing themes through interpretive engagement with the data.^67^ The development of themes and subsequent analysis was shaped by the researcher’s positionality, contextual sensitivity, and a commitment to centering participants’ voices, alongside sustained engagement with the data and relevant literature on midwifery in India.

In the findings section below, the first sub-section presents insights from the document analysis, and the second sub-section presents findings from the in-depth interviews.

## Findings

### Section 1: Findings From the Qualitative Document Analysis

This section analyses the midwifery guidelines, detailing how it frames maternal healthcare issues, the need for midwifery care, the nature of midwifery practice, and the current and proposed roles of midwives in the health system. It highlights the concerns prioritised, acknowledged, and overlooked in the guideline, bringing forth the dominant, marginalised, and absent discussions within the document.

#### Need for Midwifery

The guidelines document builds its rationale for a midwifery cadre in the public health system by anchoring it in the problem of high maternal mortality rates (MMR). It points out that the major cause of maternal deaths is poor intrapartum care caused by (1) a shortage of skilled medical staff and (2) over-medicalisation of the delivery process.^6^^(p.1)^ It highlights the increase in c-section rates as a consequence of the over-medicalisation of pregnancy and childbirth and points out the global evidence on the effectiveness of midwifery care in reducing maternal deaths and over-medicalisation. It further explains the significance of institutional delivery in reducing MMR, while drawing attention to how increasing disrespect and abuse of women during childbirth act as a deterrent to the utilisation of institutional care. However, it does not list any reasons for increased c-sections and disrespect/abuse. Thus, the guidelines focus on the immediate causes, such as a lack of staff and increased c-section.

#### History of Midwifery

The guidelines in its section on ‘history of midwifery’ chooses to begin tracing the history of midwifery from the ‘Nurse Practitioner in Midwifery’ course initiated in 2000–2003 and further outlines other attempts at formally introducing NPM programmes by the Indian Nursing Council in some Indian states.^6(p.4)^ It leaves out the history of traditional midwifery in India, the attempts to train traditional midwives as TBAs during the 1980s–90s, and the role of ANMs who worked as community midwives until they were converted to multipurpose workers over the period of 1990s to 2000s. It only recognises NPMs trained through international institutional collaborations as ‘formal’ or ‘professional’ midwives.

#### Placement of Midwives

The guidelines recognise the importance of having midwives at multiple levels of the health system by pointing out the “disparities in distribution of (maternal) deaths based on socio-demographic variables such as education, socio-economic class, caste as well as differential geographies’’ and the need to reach “remote areas including pockets of high home delivery rates and urban slums.”^6(p.1-9)^ However, the scope of work for NPMs in the guidelines is imagined mainly in secondary and tertiary levels of the health system. It states that midwifery care will be provided through ‘Midwifery-led Care Units’ established at ‘LaQshya’ certified (LaQshya is labour room quality improvement initiative. The certified health centre would have quality labour room services to deliver respectful maternity care) high caseload public health facilities with a minimum of 4 and a maximum of 18 midwives based on the caseload, at medical colleges, district hospitals (DH) and sub-district hospital/community health care facilities (CHC) in the first phase. In the second phase, “NPMs *may be* posted at 24 × 7 PHCs/ Urban PHCs remote locations with high home deliveries/ for a maximum of 3 years, following which they would be posted back at the midwifery-led care units” (emphasis added).^6(p.15)^ It explains that placing NPMs at high caseload facilities would ensure no skill loss. The guidelines repeatedly mentions the usefulness of midwifery care at ‘high case load’ facilities to ‘decongest’ them, while primary health centres are mentioned once in the entire document, and the midwives working in ‘community settings’ are mentioned in passing as part of the ‘general competencies’ (i.e., midwife’s accountabilities as a health professional). Thus, as framed by the guideline, the overall preference for midwifery care seems to lie in higher facilities and within the ambit of institutional delivery.

#### Working in Tandem

The novelty of the guidelines lies in its recognition of flaws like the lack of clear role definition and scope of work for midwives, issues around career progression, and the legal and regulatory framework in the previous NPM programmes. It emphasises the role and boundaries of midwifery multiple times, stating that “pregnant women identified with complications will be referred to a medical officer”, “midwifery-led care encourages task shifting from doctors to midwives in conduction of physiological normal births”, “only eligible women will have access to midwife-led care”, and that “NPMs will only provide midwifery care and not be rotated to other areas of nursing care.” ^6(p.10-11)^ It is understandable that since this is a midwifery guideline, the focus is on what NPMs can and cannot do; however, it does not elaborate on how the other medical staff are to work in collaboration. It does state that “midwives may also work inter-professionally; with doctors, nurses, and other health care providers as part of a maternity care team”, but it is not clear what kind of collaboration and if there will be or should be any rules issued by states to be followed while working inter-professionally. The guidelines document acknowledges the need to “develop an enabling environment for integration of NPMs” and for the “sensitisation of obstetricians, paediatricians and medical officers as well as sensitisation of field functionaries on roles and responsibilities of the midwifery personnel”, but the actual implementation may be a challenge, and the guidelines document currently does not specify those details.^6(p.10,16,17,35)^

#### Scope for Autonomous Midwifery

In the section on ‘Regulatory framework,’ the guidelines state that “the ultimate aim of the regulation is to allow nurse-midwives to practice autonomously and provide a full range of midwifery care efficiently.”^6(p.18)^ The guidelines repeatedly emphasise that “the NPM will be responsible and accountable for her practice” and describe the cadre as ‘independent.’^6(p.5,11,16)^ It leaves the work of defining what ‘autonomous’ and ‘independent’ legally mean to the National Nursing and Midwifery Commission Act (NNMCA), 2023. Despite these gaps, this imagination of the NPM provides an opportunity for midwifery advocacy in building an autonomous cadre.

In sum, the guidelines document acknowledges the problem of maternal mortality and over-medicalisation, builds a strong case for a professional midwifery cadre and draws attention to the importance of training, career progression, and clear roles, which are hallmarks of professionalisation. However, the boundaries of professional territories concerning other medical professionals who manage childbirth remain unclear. It also acknowledges issues of health personnel and the increase in c-section rates and impresses on the importance of institutional delivery and task-shifting. Thus, while the discussions on institutional deliveries and task-shifting childbirth responsibilities are dominant, the focus on the autonomy of midwives and midwifery at the primary healthcare level is limited, leaving scope for further development. One of the important silences in the guidelines is about the history of midwifery, which is notable given the way midwifery has been historically perceived and treated within the health system.

In the next section, we look at the findings from the in-depth interviews. Participants raised concerns over the position, autonomy, and placement of NPMs in the health system. Complementing the findings from the document analysis, they addressed some of the guidelines’ silences, voiced their experiences of working interprofessionally and even made some recommendations for strengthening midwifery in India.

### Section 2: Findings From the In-Depth Interviews

#### Carving out Midwifery as a Separate Profession

Given that all nurses are registered midwives in India, the participants grappled with what midwifery is and what separates staff nurses or obstetric nurses from NPMs. A midwifery trainer explained how NPMs are different from Registered Nurses and Registered Midwives (RNRM) and why a separate midwifery training is essential,

*“I think the problem is that the training has been diluted, so when the nurse qualifies, she also qualifies as a midwife, and that experience is not enough. They need separate training because they may call themselves nurse-midwives, but they have not conducted births; a lot of them have not even been into the birth process. And it is not about just holding the baby; it is about caring for mothers holistically.”* (I, Midwifery Practitioner and Educator)

Some participants expressed their discontent with calling midwives ‘nurse’ practitioners in midwifery, suggesting that it emphasised their roles as nurses over midwives, potentially subsuming midwifery within nursing. They also advocated for a 4-year direct-entry midwifery course to reinforce a separate profession.

*“Why NPM? Why not Midwives? If I call you a Medical Practitioner of Obstetrics, how would you feel? Or a Medical Practitioner of Nursing or Midwifery Practitioner of Nursing- it does not make sense. It is a separate thing; it is an established profession. We have to move forward, and direct-entry midwifery is one of those ways.”* (M, Midwifery Practitioner and Researcher)

Others further reflected on the long-standing tension between nursing and midwifery, the treatment of midwifery as a nursing specialisation, and the need to redraw boundaries, freeing the midwifery profession of its traditional connotation. The below quote suggests how the traditional overtones of the ‘Dai’ identity still seemed to percolate and affect the present understanding of who a midwife is.

“*There is a fear of the unknown, and they (nursing leadership) feel that power will go down if midwifery branches out. I always advocate for strong nursing governance by nurses and midwifery governance by midwives. It cannot be a case where medical practitioners rule nursing and midwifery, and similarly, the nursing personnel rule midwives by saying, ‘Hum bhi toh midwife hai’ (We are midwives too). I understand that nursing even after fighting to its nail, only now has kind of achieved a status as a symbol in India. But midwifery is still new, and it is a profession which people draw parallels with a ‘Dai’. Once the office accountant called me a ‘Dai’ saying that ‘for a Dai you earn quite a bit.’”* (M, Midwifery Practitioner and Researcher)

The importance of legal protections and regulatory frameworks to establish separate, independent midwifery was raised multiple times. Participants emphasised the need for a legal framework that clearly defines midwifery personnel’s role and scope of work. Providing for separate registrations, separate remuneration, allotting appropriate postings, and other clinical permissions to admit, refer, and administer drugs. They also emphasised the importance of support from other medical professionals that would enable NPMs to work fearlessly and independently. One of the participants elaborated on this,

“*There are three parts to this (establishing midwifery cadre)- one is creating a post, and the second is the lack of a legal framework. The third is support from other medical professionals, like doctors, which is missing. Therefore, there is a need for political will as well as a legal support system. Training is not the end. If we want a successful implementation, then there is a need for legally sanctioned practice.”* (E, Member of Midwifery Association)

#### Midwifery at Primary Health Care Level

There were two perspectives among participants regarding the placement of NPMs in the health system. While some felt that they were either better placed or should eventually be placed at the primary health facilities, others felt that the placement preference towards high caseload facilities was important to avoid loss of skills after midwifery training, as primary-level facilities may have fewer childbirth cases to attend to.

Some participants contested the caseload argument, drawing from their previous experiences of pilot midwifery programs that demonstrated the usefulness of midwifery at the periphery in addressing accessibility issues. One of the participants recalled,

*“In one of the Block Primary Health Centres (in a district in eastern India), every day, 30 deliveries were being conducted. We need more professional midwives at the periphery, not at the city or tertiary hospitals. Because people are not getting medical facilities, they are only going to the untrained ‘dais’ or having the normal delivery at home, assisted by family members. They want midwives at tertiary levels not only because of caseload but also because specialists are there, and they can supervise and train them.*” (D, Member of Midwifery Association)

While tertiary and high-caseload facilities were considered appropriate postings for building skills and practice, some participants explained how placing NPMs at tertiary levels can have a bearing on their autonomy and competence.

*“I am not in favour of higher facilities like medical colleges because if we place them (NPMs) there for a long time, their competency will be lost because more doctors are there, interns are there, different categories of students are there. They will take over and our NPM will be just standing and observing. In the lowest facilities they will get the chance to update their competency and skill and they will also get more opportunities to practice and if there is an emergency, she will manage and do a referral.”* (B, Member of Midwifery Association)

Some participants viewed the placement of midwives as a strategic decision. They felt that placing the newly trained midwives along with obstetricians could be tactical and help build allyship, mollifying any potential antagonism between the professions.

*“People are scared that these midwives will botch up normal births and births will be abnormal. The cabinet can go and put a freeze on ideas and growth if something goes wrong and we need to be careful. So another way of looking at it is if you can build the partnership between doctors and midwives early, then at least you have eliminated the threat. But if you go in or operate somewhere in a remote corner without them, then they might retaliate or hit back when they feel a loss of territory because there is going to be a loss of professional territory.”* (S, Obstetrician and Public Health Practitioner)

#### Creating an Enabling Environment

All participants emphasised the need to sensitise obstetricians and nurses to facilitate the successful integration of midwifery into the health system and create an enabling environment. Several participants emphasised that as midwives take on greater professional responsibilities, the support of obstetricians, both post-referral and in fostering midwives’ confidence, was crucial, highlighting the need to move beyond the traditional dichotomy wherein midwives manage normal births and obstetricians handle complications.

“*So once there is a complication, midwives have to manage it initially, and then at some point, they decide that ‘I’m not going to manage it any further. I am going to refer the woman to another level.' So, this whole notion that the midwives can do the normal and then the doctors or the specialists will do the abnormal is very flawed. Things become abnormal while you are watching, and you cannot just throw the patient across. You have to manage them, stabilise them, and shift them”* (S, Obstetrician and Public Health Practitioner)

However, nurse-midwifery leaders explained that support and collaboration from other medical professionals were difficult to achieve, citing examples of pushback from doctors, staff nurses, and other hospital staff, such as refusing timely cooperation, shifting blame for complications, and questioning their authority in the maternity ward. In the following quote, a participant highlights how an NPM seeking clinical teamwork is perceived as challenging the established norms, unlike traditional nurses who do not question the existing hierarchy.

“*Team is not available here. Even though four… four paediatricians are there. If you tell them (ask them to come), they will get angry and start scolding. ‘Why are these midwives saying all rules and responsibilities.’ Staff nurses, they will not ask. Staff nurses are going on…they will send the mothers for C-section*” (G, Nurse Practitioner in Midwifery)

To avoid pushback, nurse-midwifery leaders explained the need for clear regulations or government orders to clearly define the roles and responsibilities of midwives and other medical staff working collaboratively. They stress that without such official documentation, midwives are left to continuously assert and draw their professional boundaries and delineate their scope of work, which can be exhausting and demoralising.

“*Government should send us GOs (government orders). What is midwifery work? What is the role of the midwife? There is no GO, there is no circular. ‘We are the midwives, we have to work. We are not the staff nurses’ – we are going on explaining. Nursing and medical superintendents are changing. So, whoever comes, we have to explain everything again. The government has to send the roles. ‘If any obstetrical emergency all team should attend’- like that they have to send a circular to the paediatricians, anaesthetists, gynaecologists from the higher authority.”* (T, Nurse Practitioner in Midwifery leader)

Besides dealing with other medical personnel, midwives also encounter problems with existing administrative and institutional practices of institutional delivery. Participants explained their discomfort with incentivised target setting to increase normal birth, which goes against the idea of continuity of care.

“*You conduct more than 20 births, ten thousand (rupees) incentive. Less than 20 means five thousand. How is it possible, mam? From morning, I will be under duty. And mother will come in the morning with 2 cm (dilation). Up to two o’clock, I will monitor that mother. Every service I will provide. After two o’clock, maybe seven o’clock, or eight o’clock, she will give birth. Second midwife will write her name. Whatever I did- the morning services, it all will go, no?*” (T, Nurse Practitioner in Midwifery leader)

Participants also expressed concern over the limited understanding of midwifery among other medical professionals, noting a prevalent misconception that nurses could be equipped to perform midwifery tasks through brief skill-based training. They argued that this undermines the depth, distinctiveness, and professional scope of midwifery expertise.

“*They (medical professionals) were saying if a nurse is taught how to suture and taught how to give that perineal support, the importance of antenatal exercises, then nurses can do better, and referrals will reduce. In fact, we were told about a short-term training of 15* *days for other nurses in alternative birthing positions and respectful care*.” (R, Maternal and Child Health Consultant, NPM Programme).

## Discussion

The findings of this study highlight several key points: the tensions surrounding midwives’ autonomy, the struggle within the health system to recognise midwifery as both a form of care and a profession, and the critical need to create an enabling environment for midwives to work independently and collaboratively with other medical personnel. Central to these dynamics is the work of professional boundary drawing articulated not only by participants but also embedded within the midwifery guidelines, which together construct and legitimise the role of the NPM cadre within the health system.

### Drawing Professional Boundaries for an Autonomous Midwifery

Participants draw boundaries at three levels: a) while working with other medical professionals, b) with institutional understandings of midwifery and c) with historical and traditional perceptions of the profession.

#### Boundaries With Other Medical Professionals

Within the sociology of health and medicine, professionalisation and the social construction of professional identities have received sustained scholarly attention.^68,69^ A key focus in this body of work is how professional projects assert control over knowledge and work, protecting themselves from competing professions by constructing intra-professional and inter-professional boundaries using legitimising discourses.^70,71^ Gieryn (1983) famously argued that the aspiration to maintain professional autonomy most often involves ‘boundary work’ against threats from competing professions as ‘expansion, monopolisation and protection are generic features of professionalisation.’^72^ ^(p.792)^

While the feminist critique of the obstetric model of care elaborates on the territorial conflicts between obstetricians and midwives,^17,19,73^ the ideological and operational tensions between midwifery and the nursing profession appear more immediate and complicated as observed in some contexts.^74–76^ In India, nursing has been neglected, marked by minimal legal and administrative reforms and inadequate representation in governance structures, resulting in its continued institutional marginalisation within a doctor-dominated health system.^50^ While formal institutional integration within the nursing profession lends midwifery professional legitimacy, the fragmentation within the nursing profession through a specialised cadre increases competition between its segments. Given that midwifery has been an integral but diminished part of nursing for so long, redefining midwifery as a new cadre may cause confusion and create false competition for policy attention. Participants noted that midwifery is often an afterthought in legal and regulatory amendments, necessitating a revisit to policy regarding finance, administrative, and legal reforms within both nursing and midwifery.^77,78^ The creation of a specialised NPM cadre within nursing introduces tensions around midwives’ aspirations for autonomy, as emerging professional identities through the processes of specialisation, diversification, or substitution inevitably generate competing professional projects that jostle for occupational jurisdiction.^79^ In the case of the NPM cadre, this jostling is fuelled by two aspects: a) the nature of specialisation and b) the description of NPM as an ‘independent’ cadre. Unlike other nursing specialisations that build on a basic nursing degree, the NPM cadre embodies new knowledge and skills, raising questions about training disparities and hierarchical structures. Moreover, the policy guidelines positioning NPM as a cadre capable of practising ‘independently’, further distinguishes them while keeping them within the nursing fold. This reflects the construction of professional territories within nursing, creating ambiguity over midwifery’s jurisdiction.^12^ The dual existence of NPMs as specialised nurses and independent midwives blurs inter-professional boundaries.

The process of carving out an autonomous midwifery profession, or in other words, the process of professionalisation itself, involves marking boundaries and drawing occupational jurisdictions, requiring continuous negotiations that occur in specific historical, socio-cultural and political contexts.^12,13^ Carmel (2006)^80^ compares these boundaries to tectonic plates, where roles shift, and boundaries become less clearly demarcated. Especially in cases where professional roles overlap, different professionals perceive themselves as skilled in similar tasks and even learn on the job a craft version of the profession’s ‘knowledge systems.’^12,80(p.65-66)^ Participants observed that medical professionals frequently perceive midwifery as merely a set of transferable skills, with requests for brief training in midwifery skills reflecting a process of ‘workplace assimilation’ and ‘blurring of jurisdictional boundaries’,^81^ wherein other medical professionals attempt to encroach upon the occupational jurisdiction of midwives. The discourse on risk further complicates matters. Boundaries between obstetricians and midwives often hinge on the categorisation of ‘normal birth’ and ‘abnormal birth’, which remain porous and often shift at the operational level. This requires negotiations and adaptations involving collaborations and confrontations,^71,80,82^ reflecting territorial behaviour and cooperation.^83^

#### Institutional Understandings of Midwifery

The management and production of services are shaped by the experiences, traditions, and expertise of those involved, often resulting in routinised practices and the emergence of structural boundaries between actors.^84^ In this context, attempts to innovate service delivery, such as integrating midwifery, entail modifying these boundaries, which are constrained by policy legacies and perceived threats to professional identity.^16,85^ Participants highlight their efforts to negotiate boundaries with institutional perceptions of midwifery and utility-focused views.^86,87^ The framing of midwifery as a nurse specialisation, as additional competency, or as a collection of cost-effective skills useful in correcting the unintended consequences of existing MCH service delivery,^6(p.1-3)^ reflects a limited and reductive understanding of midwifery. As a nascent cadre, NPMs are situated within pre-existing institutional and professional hierarchies, relying on more established medical personnel to perform institutional work. This exposes them to existing hierarchies and power dynamics that influence how maternal healthcare service is organised and delivered.^84,85^ For instance, the centrally sponsored Janani Suraksha Yojana (JSY) scheme (2005), which incentivises institutional births as a strategy to reduce maternal mortality, prioritises childbirth care in secondary and tertiary institutions where dominant professions like doctors and nurses hold stronger professional territory.^11,38,88^ While the institutional delivery policy has aided in reducing maternal mortality,^89,90^ in the context of inadequate primary-level services, it has inadvertently contributed to overcrowding of tertiary facilities, over-medicalisation and promoted a more technocratic service provision affecting the professional territory of non-interventionist care.^91,92^

While the notably high C-section rates have become a rallying point for midwifery advocacy, the unintended effects of institutional delivery policies are insufficiently examined. Findings show that target-based approaches that incentivise institutional births have been incorporated into the midwifery program, assuming these methods will ensure quality care. These policy legacies, often designed with other medical personnel in mind, may undermine midwifery principles of continuity of care. This creates potential tensions between institutional demands and the needs of the women supported by midwives.^16,93^ As a result, midwives must navigate these conditions and assert their professional identity in daily practice, aligning and negotiating with the prevailing norms of maternal and newborn care.

#### Forging Identity Boundaries -Distancing From ‘Dais’ History

In delineating the professional role and identity of the NPM cadre, participants engaged in discursive and symbolic strategies through which professionals demarcate their scope of practice, domains of knowledge, and occupational legitimacy.^72^ While the NPM cadre affords a distinct identity within the nursing profession, it is a more Westernized term shaped by international standards, garnering relative credibility and respectability. As opposed to the ‘Dai,’ a figure excluded from formal recognition despite being a more locally recognisable form of midwifery in the Indian context.^22^ The findings show that NPMs navigate the dominance of technocratic care, define professional boundaries within nursing, and evade traditional and caste-based perceptions.

Much of the demand for a direct-entry midwifery course and a legal act that outlines the scope of work, rights, and protections stems from the desire to establish midwifery as a skilled and respected profession distinct from its traditional connotations and the more modern attachment with nursing. Participants’ discomfort with being referred to as ‘Dais’, and the tension around midwifery’s hierarchical position within nursing, steer midwives to seek a clear break and be distinguished as an independent profession.

### Role of Policy Documents in Drawing Professional Boundaries

Participants identified three key policy and administrative documents critical in the professionalisation process: the midwifery guideline, the National Nursing and Midwifery Commission Act (NNMCA) (2023) and government orders (GOs). While these documents assist midwives in negotiating their roles and professional boundaries, there may be other significant documents that remain unacknowledged by the participants. Rather than being static texts, documents are dynamic and socially embedded artefacts that function as central tools in the production and organisation of knowledge in modern institutions, playing a critical role in bureaucratic, legal, and organisational contexts.^94^ In occupational contexts, documents are pivotal in producing official narratives and shaping professional boundaries and identities.^95^ Medical guidelines, in particular, are instrumental in establishing and maintaining professional standards and practices.^13^ Green (2011) conceptualises documents produced in development practice as ‘living documents’ that are constantly worked upon by multiple stakeholders and, through their iterations, actively construct development categories that influence policy.^96^ This understanding of the ‘official documents’ as products of consensus-building processes is akin to the idea of policy texts being ‘boundary objects’ that facilitate collaboration by bridging gaps between communities, policymakers or varied professions.^82,97^ Boundary objects serve as interfaces between different social worlds, enabling communication and cooperation despite different practices and frameworks of understanding. Within healthcare, they allow different professionals to interact, coordinate their actions, and translate ideas while maintaining their unique identities and jurisdictional boundaries.^98–100^

The midwifery guidelines is one such socially produced document that underpins the establishment of the NPM cadre demarcating its rationale, purpose, and roles. The QDA findings reveal a dominant focus on task shifting and institutional deliveries, while discussions on professional autonomy and primary health care receive limited importance and even less reflection on the history of midwifery in India – thereby shaping the professionalisation of midwifery in specific ways. As noted earlier, institutional delivery programs can have unintended consequences in the context of inadequate staff, resources, infrastructure, and poor accessibility together contributing to over-medicalisation and disrespect and abuse in varying degrees based on the socio-economic status of women seeking care.^47,91,101^ The guidelines, however, address over-medicalisation, disrespect and abuse, staff shortages, and institutional delivery as discreet concerns without acknowledging how institutional delivery programs may further these systemic problems. This approach allows the incorporation of midwifery care within the institutional delivery framework without critically examining the inter-connections between over-medicalisation, institutional delivery, and accessibility, potentially overlooking broader associated issues.

Earlier pilot midwifery programs in West Bengal, Gujarat, and Andhra Pradesh favoured primary health centres for NPM placements.^102–104,113^ However, the 2018 guidelines prioritise decongestion and task-shifting at secondary and tertiary levels – sites of frequent medical interventions – to address over-medicalisation.^6(14-15)^ Although literature emphasises the need for resources and services at the primary healthcare level,^105–108^ participant perspectives differed on the need for midwives at the primary health facilities resembling the limited discussion on the same within the guideline. Participants’ support for positioning at high caseload facilities reflected both practical concerns of potential skill loss and strategic efforts to build alliances with other medical professionals. In this context, the guidelines, function as a consensus-building document and a ‘boundary object’ balancing clinical support for midwives and the imperative of inter-professional cooperation amid competing professional interests.^82,97^

A key omission in the guidelines document is the absence of references to traditional midwives and earlier midwifery cadres in India, mirroring participants’ discomfort with comparisons to TBAs or ‘Dais’ or even ANMs. By not acknowledging these antecedents, the guidelines implicitly distinguish between ‘professional’ or ‘formal’ midwifery and other forms of midwifery, effectively repositioning midwifery from its community roots to a more institutional framework. This omission may be an effort to side-step caste-based associations of ritual pollution linked to ‘Dais’,^9,20^ thereby framing the current cadre in a manner that aligns with biomedical legitimacy and garners respect within the broader medical community. Thus, both the study participants and the guidelines document itself draw professional boundaries distancing midwifery from its traditional context.

To promote effective collaboration and successful integration of midwifery in the health system, midwifery advocates and policymakers emphasise the importance of sensitising all involved stakeholders. Vedam et al. (2023) underscore the importance of addressing structural and professional challenges in integrating midwifery care in India through a focus on four domains: building professional identity within a new cadre, strengthening midwifery education, addressing interprofessional issues, and ensuring health system readiness. Central to these efforts is codifying a distinct scope of practice, supported by firm regulatory guidelines, autonomous roles enabling consultation and collaboration, and flexible clinical parameters that adapt to community needs.^109^ These elements resonate strongly with participants’ demands for structural clarity and institutional support to foster collaboration and minimise inter-professional tensions. While the guideline does not elaborate on the nuances of collaborative practice, a more recent document outlining the scope of practice for midwifery educators and NPMs delineates the specific competencies they may undertake independently, collaboratively, and those requiring referral.^110^ While this affords some clarity, participants desired documents that *enforced* a clear scope of work for all professions involved in collaborative care – such as nurses, obstetricians, paediatricians and other medical officers. They specifically advocated for the issuance of ‘Government orders’ (GOs) – administrative instruments that delineate the scope of work for all parties, thereby preventing territorial disputes and resistance to midwives’ role. In this context, GOs and legal acts function as ‘boundary objects’,^82,100^ structuring inter-professional relationships and facilitating the operationalisation of collaborative care by mediating between professional domains and establishing a shared framework of practice.

Participants emphasise that enabling midwifery practice requires more than individual or programmatic interventions, necessitating institutional and systemic transformation which addresses perceptions of midwifery, institutes necessary regulations, provides legal protections and establishes a clear professional identity. Replacing the Indian Nursing Act (1947), the recent NNMCA (2023) addresses some of these issues, accounting for previous critiques of not identifying midwifery or NPM as a separate cadre.^50,111^ While the revised act acknowledges and defines the terms midwifery, midwifery professionals and NPM separately in the document, it clearly states that “nurse practitioner in midwifery means a *licensed nurse* who has completed a formally recognised advanced education and training programme…” (emphasis added).^112^ This framing leaves little room for a separate, independent midwifery cadre, instead subsuming it within the nursing field.

Thus, the policy documents discussed above define, shape, and delineate the midwifery profession in various ways, acting as boundary objects that allow complex negotiations of roles, identities, and institutional power. As midwives strengthen their professional territory within the Indian health system, such negotiations will be crucial in balancing autonomy, collaboration, and integration within existing healthcare structures to improve maternal and newborn care as envisioned in the guidelines.

## Conclusion

This study highlights the role and position of midwives as envisioned in the guidelines for midwifery services in India, which frames midwifery as a specialised form of nursing care within the institutional delivery framework while distancing it from historical connotations. The findings emphasise the importance of recognising midwifery as a comprehensive profession, the need for legal and institutional support, and a reassessment of policy legacies for successfully integrating NPMs into the health system. This paper reveals the tensions surrounding midwives’ autonomy, institutional recognition, and the complexities of integrating midwifery into a medicalised care framework. The study builds on the concepts of ‘boundary work’ and ‘boundary object’ to elucidate the processes of ‘drawing professional boundaries’ embedded in policy documents and participants’ experiences, thereby unpacking the midwifery professionalisation process in the Indian context. Given the exploratory nature of this study, this paper delves into the formative processes and the negotiations midwives engage in en route to professionalisation. As the process of midwifery professionalisation progresses, as it already has, it would be interesting to further study the redrawing of professional territory and boundaries in the Indian context with more focus.

## Supplemental Material

Supplemental Material - En Route to Midwifery Professionalisation: Contextualising Midwifery Care in the Indian Public Health System Supplemental Material for En Route to Midwifery Professionalisation: Contextualising Midwifery Care in the Indian Public Health System by Sanjana Santosh in Community Health Equity Research & Policy
