# Supplementary material for: En Route to Midwifery Professionalisation: Contextualising Midwifery Care in the Indian Public Health System
Source: Community Health Equity Res Policy. 2025 May 11;46(3):321–36. doi: 10.1177/2752535X251340760 (PMC12963469; doi:10.1177/2752535X251340760)
Supplement: Supplemental Material - En Route to Midwifery Professionalisation: Contextualising Midwifery Care in the Indian Public Health System [file sj-pdf-1-qch-10.1177_2752535X251340760.pdf]

Supplementary File 1

Table 2. Code Framework

Codes	Position of Midwifery	Need and Rationale for Midwifery Cadre	MCH care delivery issues	Professionalisation Process
Sub-codes	• History of Midwifery<br>• Hierarchy of musing and midwifery<br>• Existing Skills<br>• Nature of midwifery model<br>• Scope of work	• Policy novelty<br>• Benefits of midwifery<br>• Policy goal<br>• Respectful Maternity care<br>• Task-shifting	• Importance of MMR<br>• Overmedicalisation<br>• Primary health care<br>• Rural-urban divide<br>• Referral issues<br>• Institutional delivery<br>• Congestion	• Education and Training<br>• Sensitization<br>• International standards<br>• Independent Midwifery<br>• Placement<br>• Enabling Environment

Topic List

- Usefulness/Aim of the Midwifery guideline
- Experience/Role in Midwifery Advocacy
- Selection and Format for NPM training
- Specific experience of training
- Everyday work as a midwife and workplace
- Issues in Maternal and Child Health service delivery
- Interface of NPMs and community
- Functioning of NPMs in Public hospitals
- Expectations from the new Cadre
- Role of Civil Society Organisations
